# Role of galectin-3 in diagnosis and severity assessment of epicardial artery lesions in patients with suspected coronary artery disease

**DOI:** 10.1186/s12872-023-03310-y

**Published:** 2023-05-23

**Authors:** Ivica Bošnjak, Dražen Bedeković, Kristina Selthofer-Relatić, Hrvoje Roguljić, Ivica Mihaljević, Ines Bilić-Ćurčić

**Affiliations:** 1 Department of Cardiovascular Diseases, Internal Medicine Clinic, University Hospital Centre Osijek, J. Huttlera 4, 31 000 Osijek, Croatia; 2 Department of Pathophysiology, Faculty of Medicine, J. J. Strossmayer University of Osijek, J. Huttlera 4, 31 000 Osijek, Croatia; 3 Department for Pharmacology, Faculty of Medicine, J. J. Strossmayer University of Osijek, J. Huttlera 4, 31 000 Osijek, Croatia; 4 Department of Pharmacology and Biochemistry, Faculty of Dental Medicine and Health Osijek, J. J. Strossmayer University of Osijek, 31000 Osijek, Croatia; 5 Clinical Institute of Nuclear Medicine and Radiation Protection, University Hospital Centre Osijek, 31000 Osijek, Croatia; 6 Department for Nuclear Medicine and Oncology, Faculty of Medicine, Josip Juraj Strossmayer University of Osijek, J. Huttlera 4, 31000 Osijek, Croatia; 7 Academy of Medical Sciences of Croatia, Zagreb, Croatia; 8 Department of Endocrinology and Metabolism Disorders, Internal Medicine Clinic, University Hospital Centre Osijek, J. Huttlera 4, 31 000 Osijek, Croatia

**Keywords:** Galectin-3, Coronary artery disease, Biomarker, Chronic coronary syndrome

## Abstract

**Background:**

This study aimed to investigate the possible role of serum galectin-3 (Gal-3) levels in the diagnosis and assessment of significant epicardial artery lesions in patients with suspected coronary artery disease (CAD).

**Methods:**

This was a single-center cross sectional cohort study including 168 subjects with suspected CAD and indications for coronary angiography divided into three groups: percutaneous coronary intervention (PCI) group (N 64), coronary artery bypass graft surgery (CABG) group (N 57), and group with no coronary stenosis (N 47). Gal-3 levels were measured and the syntax score (Ss) was calculated.

**Results:**

The mean value of Gal-3 in the PCI and CABG group was 19.98 ng/ml, while in the control group, it was 9.51 ng/ml (*p* < 0.001). The highest value of Gal-3 was found in the group of subjects with three-vessel disease (*p* < 0.001). When subgroups were analyzed by Gal-3 levels (< 17.8 ng/ml low, 18.8–25.9 ng/ml intermediate, > 25 ng/ml high risk) there was a significant difference between at least two Gal-3 groups for the arithmetic mean of Syntax score (*p* < 0.001). The syntax I’s arithmetic mean at low and intermediate-risk Gal-3 levels was significantly lower than at high-risk Gal-3 levels (*p* < 0.001).

**Conclusion:**

Gal-3 could be used as an additional tool for diagnosis and severity assessment of atherosclerotic disease in patients with suspected CAD. Furthermore, it could help identify high-risk subjects in patients with stable CAD.

## Background

Cardiovascular disease (CVD) is a major treatment challenge and remains the most common cause of death worldwide [1]. Atherosclerosis is a progressive vascular disease affecting all organ systems characterized by an ongoing inflammatory response crucial for the development of this common disorder. Usually, atherosclerosis and its complications refer to the epicardial or extra- and intracranial arteries, although aorta and peripheral arteries are not excluded from this pathologic condition [2]. The challenges of treatment and the difficulties of diagnosis and follow-up of patients are the reasons for the high incidence of cardiovascular disease even in modern times.

The basis for atherosclerotic plaque development is the formation of cholesterol esters, migration of monocyte-macrophage cells, and accumulation of fibrous elements in the intimal layer of the blood vessel [3]. Rupture of the plaque promotes thrombus formation, obliteration, and occlusion of the vessels, leading to complications and adverse events such as myocardial infarction or stroke, depending on the vascular region affected [3]. The formation of macrophage foam cells originates directly from the migration of activated macrophages which secrete proinflammatory cytokines such as galectin-3 (Gal-3) [3]. Therefore, it can be assumed that Gal-3 has a dependent stimulatory effect on macrophage migration.

Gal-3 is a member of a diverse galectin family involved in many physiological and pathological processes such as inflammation and fibrous tissue formation [4] and its crucial role in normal macrophage function is also well known [5, 6]. One of the most common causes of heart failure is ischemic heart disease with atherosclerosis in the background, and since macrophages play an important role in the inflammatory process of atherosclerosis, Gal-3 may also be an important factor in the overall pathological mechanism [7].

Despite declining incidence and mortality rates of CVD, prevention remains a major challenge. Several biomarkers such as troponin (Tn), brain-natriuretic peptide (BNP), C-reactive protein (CRP), creatine kinase (CK), and its myocardial isoenzyme (CK-MB) have proven useful to assess CVD activity and are released as a result of myocardial injury, so-called ‘bystander’ biomarkers. Conversly, Gal-3 [1, 8]. Bosnjak et al. and de Boer et al. plays a causal role in the remodeling process and can be called a “culprit” biomarker, more specific for stable CAD without acute myocardial injury [9].

The question arises whether measured serum Gal-3 levels are related to the extent and severity of atherosclerotic disease or could be a possible predictor of future serious adverse events (MACE).

This study aimed to investigate the possible value of serum Gal-3 levels in the diagnosis and severity assessment of significant coronary artery lesions in patients with suspected coronary artery disease (CAD). In addition, an association of Gal-3 levels with the vessel disease extensiveness in patients with proven CAD was also evaluated thus potentially providing an additional tool for identifying high-risk patients.

## Materials and methods

### Study design

The study was conducted as a single-center cross sectional study. In order to obtain a test strength of 80% and to have significance at the level of 5% (*p* = 0.05), it was estimated that each group of patients should have encompassed 50 subjects: a group of patients with PCI, a group of patients for CABG and a control group—patients with normal coronary arteries, without obstructive or nonobstructive stenosis or plaque in major coronary arteries.

One hundred sixty-eight consecutive subjects from 2018 to 2020 were included in the study who had an indication for coronary angiography based on positive test of ergometry (treadmill or cycle ergometry) or myocardial scintigraphy suggestive of myocardial ischemia. Based on coronary status enrolled patients were divided into three groups: percutaneous coronary intervention (PCI) group (N 64), coronary artery bypass graft surgery (CABG) group (N 57), and control group (N 47). In order to avoid potential confounders all patients with conditions that may affect serum galectin-3 levels were excluded from the study: patients with non-significant stenosis of coronary artery (non-obstructive coronary artery disease), heart failure, chronic kidney disease, malignancies, diabetes mellitus, hypertension grade 2 and higher, autoimmune diseases or acute infections, and patients who had previously had an acute coronary syndrome or had undergone vascular intervention (dilatation, PCI procedure, CABG). In addition, we excluded patients who had acute coronary syndrome (unstable angina pectoris, NSTEMI and STEMI patients), those whose coronary angiography was performed due to the treatment of cardiomyopathy or valvular disease as part of preoperative preparation, and a portion of subjects who had a proper finding (false positive ergometry or myocardial scintigraphy and the presence of microvascular disease).

Laboratory tests including lipid profile and echocardiography were performed at baseline. All participants received optimal drug therapy for chronic coronary syndrome according to current guidelines from the European Society of Cardiology [10], except in cases of intolerance. Subjects without any epicardial artery disease were assigned to the control group, whereas subjects with significant stenosis of epicardial coronary arteries (> 70%, for LMCA > 50%) were presented to the heart team and were assigned to the PCI group or the CABG group, depending on the revascularization method chosen.. Syntax score was calculated for subjects with significant coronary artery disease using the online Syntax Score Calculator (http://syntaxscore.org/calculator/start.htm) [11]. Syntax score is a tool designed to help cardiologists/cardiac surgeons in the decision-making process about the best type of revascularization (PCI/CABG) required. However, the final decision is made by the heart team or single operator and is not necessarily driven by Syntax score, but by other factors such as cardiac catheterization lab equipment and/or availability of cardiac surgeons.

The hospital ethics committee approved study No 25–1:5020–7/2013 and all subjects signed informed consent.

### Measurement of Gal-3

For the measurement of Gal-3, blood samples were taken and frozen immediately after the PCI procedure. The concentration of Gal-3 in serum was measured using an enzyme immunoassay (EIA) 004110 galectin-3 (LabCorp, Burlington, North Carolina) and expressed in ng/ml. The galectin-3 assay is a diagnostic, quantitative 2-site manual enzyme-linked immunosorbent assay (ELISA) validated for use in human serum. The capture monoclonal antibody (rat IgG2a) is immobilized on 96-well plates, while the detection antibody utilizes a mouse monoclonal antibody that targets the human galectin-3 protein and is conjugated with horseradish peroxidase. The calculated overall intra-ssay coefficient of variation was 7.5%, and the inter-assay coefficient of variation was 5.4%. A serum Gal-3 concentration below 17.8 ng/ml was considered normal and set as a cutoff value [12]. Based on previous studies, galectin-3 represents a potential biomarker to identify heart failure patients with the highest risk for hospital admission or death, while not exhibiting significant variations in the serum of clinically stable patients. As our patients all had stable CAD, Gal-3 measurement was not performed prior the coronary angiography. If the intervention was done instantly, the sample was taken immediately after the intervention. The interval between coronary angiography and sample collection was < 5 min therefore it had no implications on interpretation of the results.

### Statistical analysis

Statistical analysis was performed using the SPSS program, version 17.0. The T-test was used to examine the significance of differences in mean Gal-3 values ​​between patient groups. Depending on the results of Levene's test, the t-test was applied, assuming equal and unequal variances. Analysis of the significance of differences in mean Gal-3 values ​​with respect to Syntax score risk, as well as the significance of differences in mean values ​​of Syntax I, Syntax II PCI and Syntax II CABG with respect to Gal-3 levels, was performed using ANOVA (the one-way analysis of variance (ANOVA). In the case where ANOVA indicated the existence of differences between at least two groups, Tukey’s Honest Significant Difference test (HSD) was applied. Correlation analysis was performed to determine the association between Gal-3, Syntax I, Syntax II PCI, and Syntax II CABG using Pearson correlation coefficients. Statistical significance was set at *p* < 0.05.

## Results

### Study participants

This study was conducted as a single-center retrospective cohort study. 168 subjects were included with an indication for coronary angiography because of suspected coronary disease. The control group (patients with no coronary artery stenosis present) included 47 subjects, 55.32% men and 44.68% women, while in the CAD group, there were 121 subjects, 54.55% men and 44.45% women. Based on the revascularization method, subjects in the CAD group were assigned to the PCI (N 64) or CABG (N 57) group according to the decision of the heart team, whereas the control group had normal coronary angiography findings. The baseline characteristics of the subjects are summarized in Table 1. The mean age of the subjects in the study group was 63.17 ± 8.34 years, while in the control group it was 63.48 ± 9.23 years. There was no difference between the values of serum cholesterol fractions, age or sex distribution, BMI, LVEF and renal function between 3 groups (Table 1).

**Table 1:** Baseline characteristics of subjects

Variable	Control group	PCI group	CABG group
N	47	64	57
Age (year)	63.17 ± 8.34	62.13 ± 9.81	65 ± 8.34
Gender	M 54.55%, F 45.45%	M 56.25%, F 43.75%	M 52.63%, F 47.37%
LVEF	60.32 ± 8.63%	57.62 ± 10.27%	59.48 ± 10.27%
BMI	27.35 ± 1.65	27.83 ± 1.83	28.47 ± 1.93
CrCl	75.71 ± 9.55	73.83 ± 15.27	74.64 ± 9.23
TC mmol/L	5.47 ± 1.25	5.34 ± 0.97	
TG mmol/l	1.83 ± 0.89	1.68 ± 0.56	
LDL-c mmol/l	3.5 ± 0.93	3.42 ± 0.81	
Aspirin	97%	98%	96%
Statin	88%	92%	89%
CCB	55%	33%	35%
ACEI	55%	70%	67%
BB	77%	81%	83%
Other	20%	33%	37%

*ACEi* angiotensin convertase enzyme inhibitor, *BB* beta blockers, *BMI* body mass index, *CCB* calcium channel blocator, CrCrl creatinine clearance, *F* female, *Gal-3* galectin-3, *LVEF* left ventricular ejection fraction, *M* male, *TC* total cholesterol, *TG* triglycerides. *Other* drugs that do not have IA level of evidence in treatment of stable coronary heart disease, mainly symptomatic therapy (long-acting nitrates, trimetazidime)

### Gal-3 levels

The mean value of Gal-3 in the study group was 19.98 ng/ml, while in the control group, it was 9.51 ng/ml (*p* < 0.001). There was no significant difference in the levels of Gal-3 between the PCI and CABG groups (*p* = 0.164). However, there was a difference between the control and PCI group (*p* < 0.001), the and control and CABG group (*p* < 0.001) (Table 2).

**Table 2:** The difference in Gal-3 levels according to different groups of patients

		**N**	**Mean**	**SD**	**t-test**
**Control group (0 – 1)**	0	121	19,98	9,58	*t* = 9,075^b^
	1	47	9,51	5,19	*p* = 0,000*
**PCI – CABG**	PCI	64	18,84	8,59	*t* = -1,402^a^
	CABG	57	21,27	10,52	*p* = 0,164
**Control group (1) – PCI**	1	47	9,51	5,19	*t* = -6,607^a^
	PCI	64	18,84	8,59	*p* = 0,000*
**Control group (1) – CABG**	1	47	9,51	5,19	*t* = -7,418^b^
	CABG	57	21,27	10,52	*p* = 0,000*
**Age**	26 – 60	38	20,77	10,44	*t* = 0,610^a^
	61 – 84	83	19,62	9,21	*p* = 0,543
**Gender**	1	66	19,72	9,95	*t* = -0,333^a^
	2	55	20,30	9,20	*p* = 0,740
**1VD [control group (0)]**	0	78	20,91	10,71	*t* = 1,614^b^
	1	43	18,31	6,91	*p* = 0,109
**2VD [control group (0)]**	0	97	20,54	9,82	*t* = 1,292^a^
	1	25	17,73	8,38	*p* = 0,199
**3VD [control group (0)]**	0	90	17,75	7,11	*t* = -3,652^b^
	1	31	26,46	12,61	*p* = 0,001*
**LMCA [control group (0)]**	0	98	20,25	9,25	*t* = 0,630^b^
	1	24	18,85	11,05	*p* = 0,530

T-test, ^a^Equal variance assumed, ^b^Equal variances not assumed, *statistically significant at *p* < 0,05; *PCI* percutaneous coronary intervention, *CABG* coronary artery bypass graft surgery, *VD* vessel disease

There was a moderately significant relationship between Syntax score I and Gal-3 values (*p* < 0.001). For the Syntax II score, a moderately significant relationship with Gal-3 values in the PCI group (*p* < 0.001) was observed, but not in the CABG group (*p* = 0.830) (Table 3).

**Table 3:** Correlation analysis between Gal-3 levels and Ss

		**Gal-3**	**Syntax I**	**Syntax II** **PCI**	**Syntax II CABG**
**Gal-3**	*r*	1			
	*p*				
	*n*	121			
**Syntax I**	*r*	0,415	1		
	*p*	0,000*			
	*n*	120	120		
**Syntax II** **PCI**	*r*	0,390	0,277	1	
	*p*	0,001*	0,028*		
	*n*	64	63	64	
**Syntax II CABG**	*r*	-0,029	0,103		1
	*p*	0,830	0,445		
	*n*	57	57		57

Pearson correlation analysis, *Statistically significant at *p* < 0,05, *PCI* percutaneous coronary intervention, *CABG* coronary artery bypass graft surgery

**Fig. 1:**
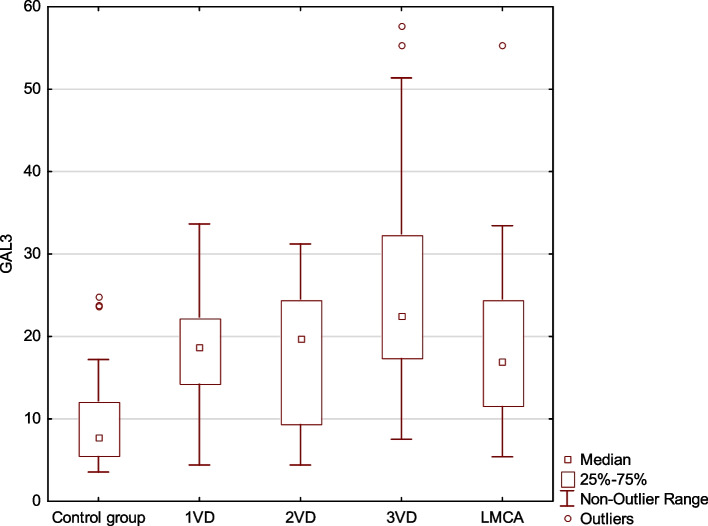
Gal-3 values in patient subgroups according to the extent of coronary artery disease

Subjects were divided into subgroups according to the number of coronary arteries affected. An increasing trend in Gal-3 levels was observed in relation to the number of vessels involved, and the highest value was found in the group of subjects with three-vessel disease (*p* < 0.001). Left main coronary artery (LMCA) involvement showed similar Gal-3 levels to single vessel disease and was classified as a special category (Fig. 1).

In addition, subjects were divided into subgroups according to Syntax score (Ss) values (Ss < 22 low risks, Ss 22–33 intermediate risk, and Ss > 33 high risks, respectively). Although there is an increasing trend in Gal-3 levels associated with elevated Ss values, statistical significance was not confirmed. When subgroups were analyzed by Gal-3 levels (< 17.8 ng/ml low, 18.8–25.9 ng/ml intermediate, > 25 ng/ml high risk) and compared with Ss values, there were significant differences between at least two Gal-3 groups for the arithmetic mean of Syntax score I (Table 4). The arithmetic means of Syntax I at low and intermediate-risk Gal-3 levels was significantly lower than at high-risk Gal-3 levels (*p* < 0.001) (Table 5).

**Table 4:** Difference between Gal-3 groups regarding the arithmetic mean of Ss I

		**N**	**Mean**	**SD**	**ANOVA**
**Gal-3 levels**	1 < 17.8 ng/ml	50	10,30	6,92	*F* = 13,898 *p* < 0,001
	2 17.8–25.9 ng/ml	48	11,19	6,19	
	3 > 25.9 ng/ml	22	19,05	7,42	

one-way ANOVA, Statistically significant at *p* < 0,05

**Table 5:** Comparison of Gal-3 risk groups regarding the arithmetic mean of Ss I

	**Compared groups**		**Mean** **difference**	***P***
**Gal-3 values**	1	2	-0,89	0,791
	1	3	-8,75	< 0,001
	2	3	-7,86	< 0,001

Tukey’s Honest Significant Difference test is statistically significant at *p* < 0,05

## Discussion

High Gal-3 levels are associated with the presence of significant carotid plaques, independent of sex, age, LDL levels, or previous myocardial infarction [13, 14]. A recent study by Li et al. indicated that serum Gal-3 levels were significantly higher in patients with angiographically proven coronary artery disease than in patients without CAD; as well as in patients with acute coronary syndrome (ACS) compared to patients without ACS [15]. Gal-3 could be an independent predictor of CAD, possibly associated with Syntax score complexity, and in the one-year follow-up, the increased risk of MACE [16, 17]. However, data on Gal-3 in patients with stable CAD are scarce and there are no studies investigating Gal-3 as a biomarker for MACE risk stratification in that subset of patients.

Our results support previous findings [17–19] confirming the association of Gal-3 levels and the presence of significant atherosclerotic epicardial stenosis, indicating that it may serve as a biomarker of a major atherosclerotic process. However, there was no difference between PCI and CABG groups in Gal-3 levels. This can be explained by the fact that the decision on the treatment modality is quite subjective, depending on the skill of the operator or invasive cardiologist as well as the available equipment. Therefore, a difference between these two groups could not be presumed because a patient undergoing PCI may have at least equally severe CAD as one undergoing CABG. In the clinical setting, the exact role of Gal-3 is not fully elucidated when it comes to coronary heart disease, but the results of other authors confirm our results [15, 20]. Furthermore, we have demonstrated the correlation between serum levels of Gal-3 and Ss I. The strongest association of Ss I with Gal-3 was observed in the group of subjects with the most complex lesions, Ss > 33. Similar results were demonstrated by Aksan et al. [21], but after adjustment for other risk factors, Ss did not prove to be an independent risk factor for the severity of lesions. On the other hand, we have avoided possible confounding factors by including a specific population without major risk factors and with no in-between group differences. Turan et al., along with other authors, have shown that Gal-3 was independently associated with Ss [22, 23]. Similar results were obtained when comparing serum Gal-3 levels with the number of vascular lesions. The highest level of Gal-3 was found in three-vessel disease, just as in the Ss > 33 group of subjects. As patients with reduced LV fraction, and significant renal impairment, were excluded from the study, it was expected that patients with lower Ss and fewer affected vessels would have lower Gal-3 values. Our findings support results from other studies reporting that patients with three-vessel disease had higher levels of Gal-3 than patients with 1- or 2-vessel disease [17, 18].

Patients with chronic coronary syndrome often develop acute coronary syndrome and other adverse events (occurrence of atrial fibrillation or heart failure) despite optimal drug therapy and nonpharmacological measures. It is necessary to identify patients from this group with the highest risk of MACE requiring invasive procedures (PCI or CABG) in addition to optimal drug therapy. In one study including patients with heart failure, the cut-off value of Gal-3 was 17.8 ng/mL, with values < 17.8 ng/mL, 17.8–23.9 ng/mL, and > 23.9 ng/mL set as low, moderate, and high risk, respectively, for MACE [12]. In our study, the arithmetic means of Syntax I was highest at high-risk Gal-3 levels. In addition, a significant correlation between Ss I and Gal-3 levels was also confirmed. Therefore, we could speculate that Gal-3 levels in combination with Ss could serve as a predictor of MACE in this subset of patients and influence therapeutic decisions.

The role of galectin-3 as a macrophage/endothelial derivative in the atherosclerotic-inflammatory process remains to be seen. Still, based on present knowledge, elevated Gal-3 levels increase the risk of plaque destabilization and the occurrence of ACS [15, 23]. Thus, patients with stable CAD, such as those included in our study, with high Gal-3 levels in addition to optimal drug therapy including statins, could be classified as high-risk patients. It should be pointed out that we did not observe any significant difference in serum lipid levels between PCI/CABG group and the control group, probably due to study exclusion criteria as well as statin subdosing of our patients. The observed association between Gal-3 and Syntax score suggests that patients with more complex and multiple lesions are at higher risk for adverse events. Other authors reported similar findings [15].

Presently, in order to prove the importance of coronary disease in our patients with angina pectoris, i.e. chronic coronary syndrome, in addition to conventional coronary angiography, we also perform additional functional tests fractional flow reserve (FFR), instantaneous wave-free ratio (iFR), diastolic hyperemia-free ratio (dFR), resting full-cycle ratio (rFR) or additional imaging methods intravascular ultrasound (IVUS) and optical coherence tomography (OCT). In this way, we confirm the hemodynamic or pathoanatomical significance of certain lesions and thereby make a decision on possible revascularization (PCI or CABG) or continuation of OMT. These are extremely expensive tests making conventional coronary angiography more costly. Identifying a valuable biomarker such as Gal-3 would be more convenient method because it is simpler, cheaper, and more practical.

We have included highly selected group of patients diagnosed with CAD only, without additional factors that might have an impact on serum Gal-3 levels. Patients in daily clinical practice are more complex and often have CAD, arterial hypertension, heart failure, diabetes, and CKD, so elevated Gal-3 levels may have an even greater significance in this group of patients for the prediction of significant coronary disease as well as adverse cardiovascular events. Determination of Gal-3 in patients with suspected coronary disease not only indicates the significance of the present coronary disease, but also stratifies them as a high-risk patients. Many patients, despite adequate revascularization and DE stents of new generations, still end up on the recoronarography, which increases the costs of the healthcare system. Such patients, in addition to having a significant coronary disease, experience MACE in the form of heart attack, stroke, TLR (target lesion revascularisation), not to mention the risk of developing heart failure, primarily with preserved but also reduced EF, which reduces the survival rate, increases hospitalization costs and impairs quality and life expectancy. Therefore, adequate stratification would help in the detection of such patients who not only need to treat coronary disease but also need to prevent potential heart failure. In addition to the importance of Gal-3 as a diagnostic and prognostic biomarker for atherosclerotic disease and heart failure, Gal-3 may also be a potential target for pharmacological treatment to inhibit inflammatory and fibrotic tissue processes.

The main limitation of this study is the relatively small number of subjects since this was a single-center cross-sectional study. Further prospective studies including a larger sample size monitoring the outcomes should be conducted to explore the prognostic value of Gal-3 in patients with stable CAD. In addition, it would be interesting to include subjects with non-obstructive coronary heart disease and coronary artery stenosis < 50, and assess the association between Gal-3 and other biomarkers such as troponin, CRP and NT-proBNP to further elucidate the underlying pathophysiological mechanism involved in CAD. However, one of the main strengths of our study is the recruitment of a patient population with “pure” CAD and no other significant comorbidities that could affect Gal-3 levels. Moreover, there were no significant differences in baseline characteristics between the groups thus avoiding potential confounding factors.

## Conclusions

The results of our study suggest that Gal-3 may be a useful biomarker in determining and assessing the severity of coronary heart disease in patients with suspected CAD. In the group of subjects with proven CVD and elevated Gal-3 serum levels, greater extensiveness of coronary heart disease (three-vessel disease) could be expected. Furthermore, Gal-3 serum levels could present an additional tool in order to identify high-risk patients with stable coronary heart disease, especially those patients who would benefit most from early revascularization regardless of whether it is CABG or PCI with optimization of drug therapy in order to prevent progression of the disease, either fibrosis, heart failure or the development of MACE.

## Data Availability

The datasets used and/or analyzed during the current study are available from the corresponding author on reasonable request.

## Abbreviations

CVD: Cardiovascular disease
Gal-3: Galectin-3
Tn: Troponin
BNP: Brain-natriuretic peptide
CRP: C-reactive protein
CK: Creatine kinase
CK-MB: Myocardial isoenzyme
MACE: Major adverse cardiac events
PCI: Percutaneous coronary intervention
CABG: Coronary artery bypass graft surgery
BMI: Body mass index
LVEF: Left ventricular ejection fraction
LMCA: Left main coronary artery
Ss: Syntax score
EIA: Enzyme immunoassay
ox-LDL: Oxidised low density lipoprotein
CKD: Chronic kidney disease
